# Higher recipient pre‐transplant FOXP3 mRNA expression is associated with acute leukaemia relapse after HSCT

**DOI:** 10.1002/jha2.438

**Published:** 2022-04-30

**Authors:** Niels Jacobsen, Tina Frisch, Niels Keiding, Carsten Heilmann, Henrik Sengeløv, Hans O. Madsen, Ebbe Dickmeiss, Lars P. Ryder

**Affiliations:** ^1^ Department of Hematology Rigshospitalet University Hospital Copenhagen Denmark; ^2^ Department of Clinical Immunology Tissue Typing Laboratory Rigshospitalet University Hospital Copenhagen Denmark; ^3^ Department of Biostatistics Faculty of Health Sciences Copenhagen University Copenhagen Denmark; ^4^ Paediatric and Adolescence Medicine Rigshospitalet University Hospital Copenhagen Denmark

**Keywords:** acute leukaemia, FOXP3, haematopoietic stem cell transplantation, HSCT, Treg cells

## Abstract

The effect of higher FOXP3 mRNA expression by recipient pre‐transplant CD4+ T cells on leukaemia relapse was analysed in a series of 106 patients who received allogeneic haematopoietic stem cell transplantation after myeloablative conditioning with or without antithymocyte globulin (ATG) due to acute leukaemia in 1st or 2nd complete remission. FOXP3 mRNA was measured by qPCR in purified CD4+ T cells from blood obtained before conditioning. Higher FOXP3 mRNA expression was associated with an increased relapse risk when conditioning included ATG (*n* = 43, hazard ratio [HR] 11.0 [2.50–48.4], *p* = 0.00001). No effect was observed in patients not receiving ATG (HR 0.95 [0.53–1.81]).

## 1 INTRODUCTION

Relapse of acute leukaemia is a major cause of treatment failure after allogeneic haematopoietic stem cell transplantation (HSCT) [1].

CD4+CD25+FOXP3+ regulatory T cells (Treg cells) suppress immune reactions against auto‐ and alloantigens including tumour antigens [2]. Treg cells may accumulate in bone marrow and blood from patients with acute myelogenous leukaemia (AML) [3, 4] or acute lymphoblastic leukaemia (ALL) [5]. However, whether patient Treg cells suppress anti‐leukaemia immune reactions and contribute to leukaemia progression is not clear.

Expression of the transcription factor FOXP3 is essential for the suppressor function of Treg cells [6]. A higher FOXP3 mRNA expression by *donor* pre‐harvest CD4+ T cells has been associated with late relapse of ALL after HSCT [7]. We ask the question whether a higher FOXP3 mRNA expression in *recipient* CD4+ T cells obtained from peripheral blood prior to myeloablative conditioning was associated with an increased risk of posttransplant leukaemia relapse.

## 2 PATIENTS AND METHODS

The study was approved by the Committees on Health Research Ethics for the Capital Region of Denmark (H‐4‐2013‐188) and the Data Protection Agency (30‐1168). Informed written consent was obtained according to The Declaration of Helsinki.

Patient selection criteria included availability of frozen blood cells for retrospective analysis and no previous allogeneic transplantation. The study comprised 106 children and adults, median age 28.8 (3.3–55.0) years who received myeloablative, allogeneic HSCT during 1998–2006 due to AML (*n* = 49) or ALL (*n* = 57) in complete haematological remission (CR); 63 patients were in 1st CR, and 43 patients were in 2nd CR.

Donor was an HLA identical sibling (*n* = 46) or an alternative donor (*n* = 60), comprising HLA compatible other related (*n* = 2) or unrelated donors (*n* = 58) (Table S1). Median donor age was 35.0 years. Forty‐one alternative donor‐recipient pairs were typed for HLA ABC‐DRB1 and ‐DQB1 by high resolution, and 19 pairs were typed by low resolution for HLA‐ABC and by high resolution for DRB1 and DQB1. Sixteen alternative donor‐recipient pairs had at least one documented allele or antigen mismatch in the anti‐host and/or anti‐graft direction [7].

### 2.1 Transplant procedures

Myeloablative conditioning included TBI (*n* = 101) or busulfan (*n* = 5) with cyclophosphamide or etoposide [7]. Forty‐three of 60 recipients with alternative donor received additional immunosuppression during conditioning with antithymocyte globulin (ATG), either horse‐ATG (ATGAM, Upjohn), 20 mg/kg/d (*n* = 12) or rabbit‐ATG (Thymoglobulin, Merieux) 2.5 mg/kg/d (*n* = 31) for 3 days (Day ‐5, ‐4, ‐3) (Table S1). GvHD prophylaxis consisted of cyclosporine with or without methotrexate (MTX) [7]. At day 0, patients received iv. bone marrow stem cells (BMSCs, *n* = 62), or mobilized peripheral blood stem cells (PBSCs, *n* = 44).

Leukaemia relapse was defined morphologically.

### 2.2 FOXP3 analysis

CD4+CD3+ T cells obtained 1–2 weeks before conditioning were purified and analysed as described [7, 8]. Full length and the Δ2 splice variant of FOXP3 mRNA, but excluding Δ2Δ7, were analysed in duplicate in purified CD4+ T cells by a cDNA‐based qPCR system using CD4 mRNA as a population‐specific internal reference [7, 8]. The obtained ‐ΔCT value, which provided a log scale estimate of the number of FOXP3 mRNA copies relative to the number of CD4+ T cells [7], was expressed as a continuous variable in a non‐log scale unless otherwise stated.

### 2.3 Statistical analysis

Risk factors for relapse were identified by Cox proportional hazard regression and tested by Wald test. All risk factors met the proportionality assumption. Relapse was analysed by censoring patients at last follow‐up, if they died in continuous CR (CCR), or if they received a second donor cell infusion. Treatment failure was defined as relapse or death in CCR after HSCT using censoring at last follow‐up or a second donor cell infusion. Cumulative relapse incidence was analysed treating death in CCR as a competing event. Disease‐free survival was estimated according to Kaplan–Meier using treatment failure as the event, last follow‐up or a second donor cell infusion as censoring, and the Mantel‐Cox log‐rank test for significance. All *p* values were two‐tailed. *p* < 0.050 was considered significant [7].

## 3 RESULTS

Patient, donor, and procedure‐related characteristics are shown in Table S1. ATG recipients more often had alternative donor (*p* < 0.0001), HLA mismatch donor (*p* = 0.0056), received BMSC rather than PBSC (*p* < 0.0001), and **received** combined cyclosporin‐MTX GvHD prophylaxis (*p* = 0.002). Relapse occurred in 34 of 106 patients median 250.5 days (77–2606 days) after HSCT. Eleven patients died in CCR median 206 days after HSCT (16–2959 days). Three patients received additional donor cell infusion due to pancytopenia without evidence of relapse or graft rejection. Sixty‐one patients survived in CCR after a median observation time 2942 days.

In purified CD4+ T cells, the expression of FOXP3 mRNA ‐ΔCT correlated with the expression of IL‐2Rα mRNA and CTLA4 mRNA, but was independent of age within the interval tested.

### 3.1 Univariate regression analysis of relapse

Since it has been suggested that ATG mediated expansion of Treg cells ex vivo [9], we analysed the effect of higher recipient FOXP3 mRNA expression after stratification for ATG (Table 1). When conditioning included ATG, a higher FOXP3 mRNA expression was strongly associated with an increased risk of relapse (hazard ratio [HR] 11.0 [2.50–48.4], *p* < 0.00001). No similar effect was observed in the absence of ATG. No other significant risk factors were identified. Similar effects were observed in ALL and AML, despite the limited number of AML patients (Table 1). Neither a higher recipient FOXP3 mRNA expression nor ATG per se was significantly associated with relapse (Table 1, *p* = 0.38 and 0.57, respectively). Recipient preconditioning total CD4+ T cell concentration did not influence the relapse risk.

**TABLE 1:** Univariate regression analysis of relapse

Selected patient group	Stratification for ATG^¶^	Variable	┘E: n	HR (95% γCI)	P Wald
ALL and AML	ǂ 0/1	Recipient FOXP3 mRNA expression	34:106	1.20 (0.78–1.85)	0.38
	ǂ 0/1	ATG included in the conditioning	34:106	0.82 (0.40–1.66)	0.57
	^α^1	Recipient FOXP3 mRNA expression	13:43	11.0 (2.50–48.4)	0.00001
	^β^ 0	Recipient FOXP3 mRNA expression	21:63	0.95 (0.53–1.81)	0.86
Horse ATG	1	Recipient FOXP3 mRNA expression	3:12	237.2 (0.72–7779.2)	0.059
Rabbit ATG	1	Recipient FOXP3 mRNA expression	10:31	6.01 (1.09–33.19)	0.036
ALL	0/1	Recipient FOXP3 mRNA expression	20:57	1.27 (0.80–2.03)	0.30
	1	Recipient FOXP3 mRNA expression	9:26	10.32 (1.64–64.8)	0.011
	0	Recipient FOXP3 mRNA expression	11:31	1.06 (0.58–1.93)	0.85
AML	0/1	Recipient FOXP3 mRNA expression	14:49	1.01 (0.39–2.61)	0.98
	1	Recipient FOXP3 mRNA expression	4:17	57.8 (1.13–2952.4)	0.039
	0	Recipient FOXP3 mRNA expression	10:32	0.69 (0.19–2.51)	0.56
Alternative donor	1	Recipient FOXP3 mRNA expression	13:43	11.00 (2.50–48.35)	0.0012
	0	Recipient FOXP3 mRNA expression	8:17	0.80 (0.13–4.94)	0.80
HLA Match	1	Recipient FOXP3 mRNA expression	10:31	12.28 (2.62–57.53)	0.0012
	0	Recipient FOXP3 mRNA expression	19:59	0.98 (0.55–1.75)	0.945
Graft BMSC	1	Recipient FOXP3 mRNA expression	11:37	8.81 (1.745–44.51)	0.0072
	0	Recipient FOXP3 mRNA expression	13:25	0.88 (0.47–1.64)	0.67
Rec. age < 28.8 y	1	Recipient FOXP3 mRNA expression	6:26	22.89 (1.34–391.23)	0.028
	0	Recipient FOXP3 mRNA expression	8:26	1.17 (0.62–2.19)	0.61
Rec. age ≥28.8 y	1	Recipient FOXP3 mRNA expression	7:17	7.29 (1.11–47.94)	0.035
	0	Recipient FOXP3 mRNA expression	13:37	0.62 (0.18–2.16)	0.44
Conditioning with cyclophosphamide	1	Recipient FOXP3 mRNA expression	9:29	84.16 (19.06–371.59)	0.00024
	0	Recipient FOXP3 mRNA expression	13:43	0.52 (0.15–1.81)	0.29
GvHD prophylaxis Including MTX.	1	Recipient FOXP3 mRNA expression	13:43	11.0 (2.50–48.35)	0.0012
	0	Recipient FOXP3 mRNA expression	17:49	0.61 (0.18–2.00)	0.61

*Note*: FOXP3 mRNA expression was estimated as a continuous variable in a non‐log scale. The table provides estimates of FOXP3 mRNA expression relative to CD4 mRNA expression. Similar conclusions were obtained when FOXP3 mRNA was expressed per litre of blood. Median recipient Foxp3 mRNA relative to CD4 mRNA was 0.37 (range 0.049–4.36). Corresponding results for donors were 0.53 (range 0.055–3.73).

Abbreviations: ALL, acute lymphoblastic leukaemia; AML, acute myeloid leukaemia; BMSC, bone marrow stem cells; HR, hazard ratio.

¶ATG: antithymocyte globulin.

┘E: n: number of events (relapse): number of patients.

^α^ATG = 1: ATG was included in the conditioning.

^β^ATG = 0: ATG was not: included in the conditioning.

^ǂ^ATG 0/1: Without stratification for ATG.

γ: CI = confidence interval.

By restricting the analysis to patients with alternative donor, we excluded the possibility that ATG was a proxy for alternative donor. In this subgroup, the effect associated with higher FOXP3 mRNA expression was still significant provided the conditioning included ATG (Table 1). Similar results were obtained after restricting the analysis to donor‐recipient HLA match, recipient age, graft source, cyclophosphamide, or combined cyclosporine‐MTX GvHD prophylaxis. This analysis also demonstrated consistent effect of higher Foxp3 mRNA expression across heterogeneous recipient subgroups. For every subgroup, the effect of higher FOXP3 mRNA was significant, provided conditioning included ATG, but absent when ATG was not included.

### 3.2 Multivariate regression analysis of relapse

The effect of higher recipient FOXP3 mRNA expression in the presence of ATG was confirmed by multivariate regression analysis, which corrected for the effect of stage at HSCT, graft source, high‐risk cytogenetics [7] at diagnosis, and time to achieve CR on chemotherapy (HR = 9.25 [2.02–42.50], *p* = 0.0035). Measurable residual disease (MRD) data were not available. The effect of higher recipient FOXP3 mRNA expression was significant in rabbit‐ATG recipients (*p* = 0.036) and marginally significant in horse‐ATG recipients (*p* = 0.059). The low numbers prohibited conclusions regarding a possible difference regarding ATG source.

### 3.3 Cumulative relapse incidence

**FIGURE 1:**
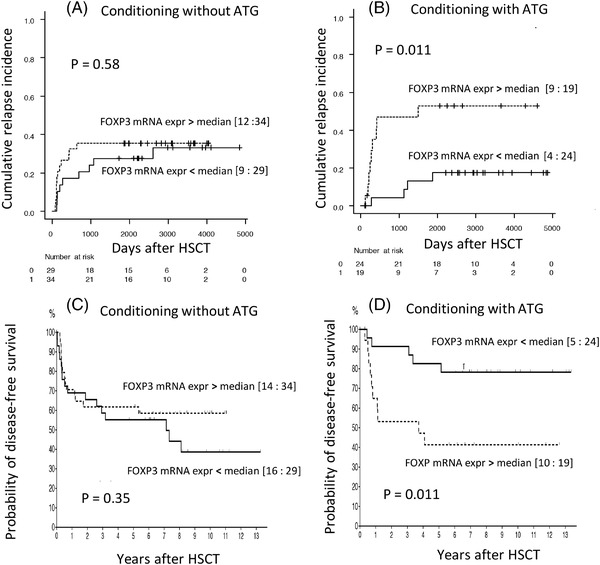
Post‐transplant relapse and disease‐free survival after haematopoietic stem cell transplantation (HSCT) for acute leukaemia. Recipient FOXP3 mRNA expression in purified peripheral blood CD4+ T cells obtained prior to conditioning was analysed as a categorial variable above (dotted lines) or below (solid lines) the median level of FOXP3 mRNA expression. (A and B) Cumulative relapse incidences after HSCT. Figures in brackets denote the number of patients with event (relapse): number of patients. (A) Conditioning without antithymocyte globulin (ATG). (B) Conditioning including ATG. (C and D) Kaplan–Meier estimates of the probability of disease‐free survival after HSCT. Figures in brackets denote the number of patients with event (treatment failure): number of patients. (C) Conditioning without ATG. (D) Conditioning including ATG. ATG, antithymocyte globulin; CCR: continuous complete remission; Expr: expression; HSCT, haematopoietic stem cell transplantation

When conditioning included ATG, and FOXP3 mRNA expression was above the median, we observed nine relapses in 19 patients in contrast to four of 24 when FOXP3 expression was below median (cumulative relapse incidence 53% vs. 17%, *p *= 0.011, Figure 1B). No difference was observed in the absence of ATG (*p* = 0.58, Figure 1A).

### 3.4 Treatment failure

The increased risk of relapse was associated with a decreased Kaplan–Meier estimate of disease‐free survival (Figure 1C,D).

### 3.5 Effect on GvHD

To demonstrate an effect of recipient FOXP3 mRNA expression on GvHD would require a larger data set.

## 4 DISCUSSION

Treg level was estimated by assessment of FOXP3 mRNA expression by qPCR in contrast to Treg cell numbers measured by flow cytometry. In mice, the capacity of Treg cells to prevent allograft rejection was more closely associated with FOXP3 mRNA expression than with flowcytometric results [10].

Support for a role of recipient Treg cells in HSCT has been provided by Inoue et al. [11] After myeloablative conditioning, murine recipient Treg cells transiently proliferate and remain immunosuppressive by inhibiting recipient dendritic cell (DC) maturation, generating tolerogenic DC unable to activate transplanted donor effector T cells, resulting in abrogation of the donor cell mediated graft‐versus‐leukaemia reaction [11].

ATG and Treg cells both mediate immunosuppression by inhibition of DC maturation and by T cell depletion. The observed interaction between ATG and FOXP3 mRNA expression suggests that complementary mechanisms may operate [11, 12]. The notion that ATG induces Treg cell expansion ex vivo has been contradicted [9, 13] and may not explain the immunosuppressive effect of ATG [14]. We confirmed the absence of an effect of ATG per se on relapse (Table 1), consistent with most clinical studies [15].

Limitation of the study: Data on MRD, a risk factor for relapse and possibly causally related to Treg cell expansion [4], were unavailable.

Conclusion: In patients with acute leukaemia, a higher recipient preconditioning FOXP3 mRNA expression in peripheral blood CD4+ T cells was associated with an increased relapse risk and a decreased probability of disease‐free survival after HSCT, provided ATG was included in the conditioning.

## CONFLICT OF INTEREST

The authors declare no conflict of interest.

## ETHICS STATEMENT

An ethics statement as been provided in the beginning of the the Result Section togather with a statement about data the confidence law compliance.

## AUTHOR CONTRIBUTIONS

*Research design*: NJ, LPR, and ED. *Laboratory analyses*: TF, HOM, and LPR. *Statistical analysis*: NJ, NK, HS, and CH. *Manuscript*: NJ.

## Supporting information

Table S1

## Data Availability

Data are available from the corresponding author.
