# Supplementary material for: Higher recipient pre‐transplant FOXP3 mRNA expression is associated with acute leukaemia relapse after HSCT
Source: EJHaem. 2022 Apr 30;3(3):975–9. doi: 10.1002/jha2.438 (PMC9421961; doi:10.1002/jha2.438)
Supplement: Supplementary file 1 — Table S1 [file JHA2-3-975-s001.docx]

Supplementary TABLE Sl. Patient, donor, and treatment related characteristics.

|  | No ATG | ATG | Total | Pχ^2^ |
| --- | --- | --- | --- | --- |
| Disease: ALL | 31 | 26 | 57 |  |
| Disease: AML | 32 | 17 | 49 |  |
| Total | 63 | 43 | 106 | 0.35 |
| Donor age <35.0 y | 30 | 22 | 52 |  |
| Donor age ≥35.0 y | 33 | 21 | 54 |  |
| Total | 63 | 43 | 106 | 0.87 |
| Recipient age <28.8 y | 26 | 26 | 52 |  |
| Recipient age ≥28.8 y | 37 | 17 | 54 |  |
| Total | 63 | 43 | 106 | 0.08 |
| Donor: HLA identical sibling | 46 | 0 | 46 |  |
| Alternative donor | 17 | 43 | 60 |  |
| Total | 63 | 43 | 106 | <0.0001 |
| Donor-recipient HLA matchⴕ | 59 | 31 | 90 |  |
| Donor-recipient HLA mismatchǂ | 4 | 12 | 16 |  |
| Total | 63 | 43 | 106 | <0.0056 |
| Stage at HSCT: 1^st^  CR | 41 | 22 | 63 |  |
| Stage at HSCT: 2^nd^ CR | 22 | 21 | 43 |  |
| Total | 63 | 43 | 106 | 0.22 |
| Bone marrow stem cell graft | 25 | 37 | 62 |  |
| Peripheral stem cell graft | 38 | 6 | 44 |  |
| Total | 63 | 43 | 106 | <0.0001 |
| Busulfan | 2 | 3 | 5 |  |
| Total body irradiation | 61 | 40 | 101 |  |
| Total | 63 | 43 | 106 | 0.66 |
| Cyclosporine alone | 14 | 0 | 14 |  |
| Cyclosporine + MTX | 49 | 43 | 92 |  |
| Total | 63 | 43 | 106 | 0.002 |

‘HLA match’ denotes the absence of documented HLA disparity.
